# The Dark Side of the Force: When the Immune System Is the Fuel of Tumor Onset

**DOI:** 10.3390/ijms22031224

**Published:** 2021-01-27

**Authors:** Elisabeth Digifico, Silvia Balinzo, Cristina Belgiovine

**Affiliations:** Humanitas Clinical and Research Center—IRCCS, Via Manzoni 56, 20089 Rozzano (MI), Italy; silvia.balinzo@gmail.com (S.B.); cristina.belgiovine@humanitasresearch.it (C.B.)

**Keywords:** chronic inflammation, tumor onset, cancer-related inflammation

## Abstract

Nowadays, it is well accepted that inflammation is a critical player in cancer, being, in most cases, the main character of the process. Different types of tumor arise from sites of infection or chronic inflammation. This non-resolving inflammation is responsible for tumor development at different levels: it promotes tumor initiation, as well as tumor progression, stimulating both tumor growth and metastasis. Environmental factors, lifestyle and infections are the three main triggers of chronic immune activation that promote or increase the risk of many different cancers. In this review, we focus our attention on tumor onset; in particular, we summarize the knowledge about the cause and the mechanisms behind the inflammation-driven cancer development.

## 1. Introduction

The main role of inflammation is usually linked to defense mechanisms to fight pathogens, on the one hand, and to repair damaged tissues, on the other. Generally, inflammation can be described as acute or chronic, depending on its duration in time. During acute inflammation, the reaction is self-limiting and when it turns off, the resolution of inflammation starts; this is an active process in which specific mediators, named resolvins and protectins, restore tissue homeostasis [1]. Unfortunately, there are several conditions in which inflammation cannot be solved and it becomes chronic. The continuous recruitment of inflammatory cells and the consequent production of inflammatory mediators finally destroy the local tissue homeostasis [2].

Less than 10 years ago, Hanahan and Weinberg established that chronic inflammation has to be considered one of the seven hallmarks of cancer [3]; evidence supporting this statement were found several years before when Virchow suggested the existence of a link between chronic inflammation and cancer [4]. 

The crosstalk between the tumor microenvironment (TME) and tumor cells is mutual; in fact, it is well known that inflammatory mediators, soluble or cellular, can be responsible for tumor initiation and progression [5,6]. Moreover, it is well accepted that tumors can also induce inflammatory reactions through the release of chemotactic factors that are able to recruit macrophages and neutrophils, to activate granulocytes via PAMPs (pathogen-associated molecular patterns) and to create an acidic and hypoxic environment [7,8].

Chronic inflammation can be caused not only by environmental factors, but also by non-resolving infections; pathogens trigger the immune system to release reactive oxygen and nitrogen species (ROS and RNS) that cause DNA damage in proliferating cells [9]. ROS and RNS are produced by mitochondria and by NADPH (nicotinamide adenine dinucleotide phosphate) oxidase, usually present in phagocytes [4,10]; for this reason, the first DNA affected by mutation is indeed mtDNA (mitochondrial DNA) [11,12]. mtDNA mutation is involved in cancer-related inflammation of several tumors, such as malignant pleural mesothelioma [13]. Moreover, oxidative damage of mtDNA, usually repaired by the base excision repair (BER) pathway [14], is significant in inflammation-associated cancers due to its link to apoptosis [12,15]. The most abundant type of DNA damage is oxidation [16,17,18]; in particular, cancer cells accumulate 8-oxoguanine with a frequency of 10^5^ per cell per day [16,19]. Additionally, several proteins, particularly DNA repair proteins, can be modified by oxidative stress via modifications on cysteine residues [20,21,22,23,24]. However, oxidation can also inactivate DNA in a non-specific way, and this modulation causes accumulation of DNA lesions, genomic instability and cancer [16,25]. 

**Figure 1:**
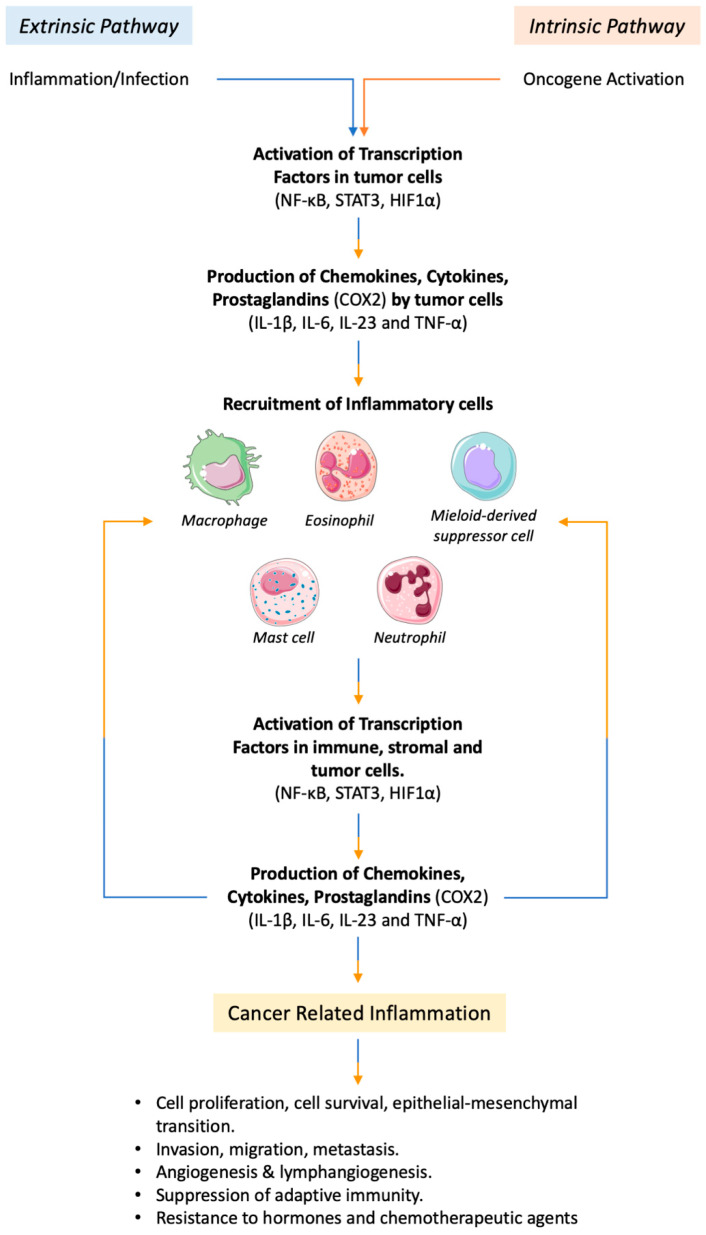
Inflammation and cancer connection pathways. An intrinsic and an extrinsic pathway connect inflammation and cancer. The extrinsic pathway is triggered by inflammatory or infectious conditions that increase tumor risk. In the intrinsic pathway, genetic alterations induce inflammation and tumor development. Although they have a different origin, both pathways converge in the activation of key transcriptional factors and inflammatory mediators, which are responsible for the recruitment of inflammatory cells. Once recruited, these cells fuel the process of cancer-related inflammation and tumor development. This figure was made with Servier Medical Art templates, which are licensed under a Creative Commons Attribution 3.0. Unported License (https://smart.servier.com).

The presence of several viral agents, such as DNA tumor viruses, can lead to tumorigenesis by direct transformation of the cells through the insertion of active oncogenes into the host genome [26]. Inflammation and cancer are linked by two pathways: the intrinsic and the extrinsic pathway (Figure 1). The intrinsic pathway is triggered by tumor-promoting genetic events, such as mutations that activate oncogenes, chromosomal rearrangement and inactivation of onco-suppressor genes. The resulting mutated cells release inflammatory mediators and generate an inflammatory tumor microenvironment, although there is not underlying inflammation. As the opposite, the extrinsic pathway is driven by inflammatory or infectious conditions that increase the risk of developing tumors in specific body sites. Both pathways can activate transcription factors, such as NF-κB (nuclear factor-κB), HIF1α (hypoxia-inducible factor 1α) and STAT3 (signal transducer and activator of transcription 3), in cancer cells. These factors in turns activate the production of cytokines (e.g., interleukin-1β (IL-1β), interleukin-6 (IL-6), interleukin-23 (IL-23) and tumor necrosis factor-α (TNF-α)), chemokines and prostaglandins that recruit leukocytes, mainly myelomonocytic cells, and further activate the key transcription factors (NF-κB, STAT3 and HIF1α) in inflammatory, stromal and tumor cells [27,28,29,30,31,32]. This chain of events leads to the generation of cancer-related inflammation hat is able to fuel tumor development [5]. Of note, Ridker et al. demonstrated that an anti-inflammatory therapy that involves IL-1β inhibition with a specific antibody can significantly reduce the incidence of lung cancer and its associated mortality in a large cohort of high-risk patients [33]. 

The TME that supports tumor onset and progression is characterized by immune suppression. The major actors in the myeloid compartment are tumor-associated macrophages (TAM); these cells are characterized by M2-like features [34] and they are responsible for the secretion of signaling molecules, such as transforming growth factor beta (TGF-β), vascular endothelial growth factor (VEGF), macrophage colony-stimulating factor (M-CSF), interleukins or chemokines (interleukin-10 (IL-10), IL-6, and C-X-C motif chemokine 8 (CXCL-8)) [35,36,37] and extracellular vesicles (EVs) with immunosuppressive properties [38]. Concerning the lymphoid compartment in the TME, T-regulatory cells (Tregs), a subset of CD4+ T cells, can induce an immunosuppressive environment favoring tumorigenesis [39,40]. Treg cells express high levels of interleukin-2 receptor (IL-2R) and consume the IL-2 (interleukin-2) produced by activated T cells, leading to effector T-cell deprivation [41,42]; moreover, they release high levels of IL-10, IL-35 (interleukin-35), TGF-β, granzyme B and perforin [43,44,45]. Furthermore, Treg cells inhibit the lymphoid function through the upregulation of CTLA-4 (cytotoxic T lymphocyte-associated antigen-4), PD-1 (programmed cell death-1), LAG-3 (lymphocyte activation gene-3), TIGIT (T-cell immunoreceptor with Ig and ITIM domains) and TIM-3 (T cell immunoglobulin and mucin domain-containing protein 3) [46]. 

**Figure 2:**
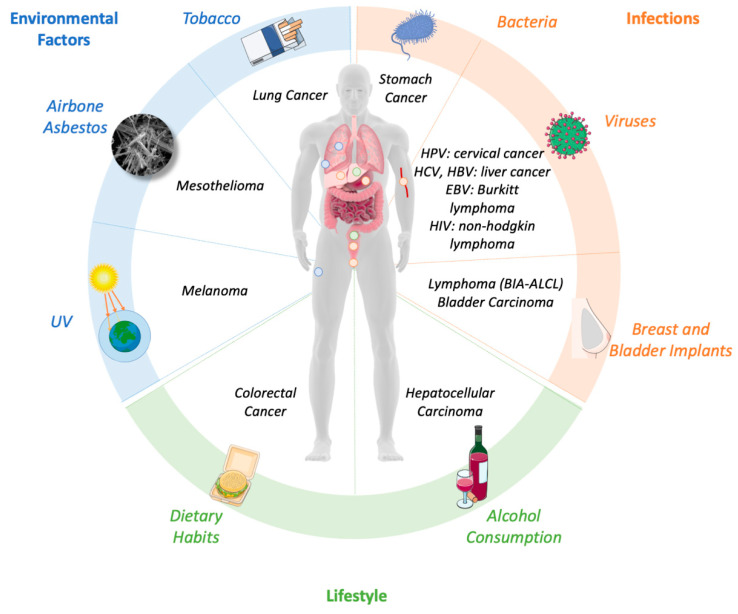
The main triggers of chronic inflammation. Environmental factors, lifestyle habits and infections induce chronic, unleashed inflammation that are able to increase the risk of different tumor types. This figure was made with Servier Medical Art templates, which are licensed under a Creative Commons Attribution 3.0. Unported License (https://smart.servier.com).

Up to 20% of all tumors arise from sites of infection or chronic inflammation; this non-resolving inflammation precedes and promotes cancer formation in those areas [5,6,10]. Environmental factors, such as tobacco smoke or asbestos inhalation; lifestyle habits such as diet and alcohol consumption, and infections, such as *Helicobacter pylori*, are the three main triggers of chronic, unleashed inflammation able to promote different cancer types (Figure 2). Here, we summarize which factors can fuel inflammation and how our immune system is pushed to go to “the dark side of the force”. 

## 2. Environmental Factors

### 2.1. Tobacco Smoke 

Tobacco smoke (TS) represents the leading cause of lung cancer worldwide. More than 85% of lung cancer deaths are due to cigarette smoke [47]. Tobacco contains about 8000 compounds, including 70 carcinogens and 20 lung carcinogens [48]. It is able to induce strong pulmonary inflammation, stimulating the release of cytokines/chemokines, finally producing reactive oxygen and nitrogen species that promote tumor onset. This oxidative stress contributes to DNA damage and induces oncogenic mutations [49,50,51]. In particular, TS stimulates inflammation by inducing the production of pro-inflammatory cytokines, such as TNF-α, IL-1 (interleukin-1), IL-6, IL-8 (interleukin-8) and GM-CSF (granulocyte–macrophage colony-stimulating factor), thus increasing the recruitment of immune cells in the airways [52,53]. A significant increase in the number of neutrophils, macrophages and dendritic cells in the airways of smokers and smoke-exposed animals compared with controls has been shown [54,55,56]. Moreover, TS-induced pulmonary inflammation promotes progressive lung destruction in Chronic Obstructive Pulmonary Disease (COPD), known to increase lung cancer risk [57,58]. 

Wang et al. demonstrated that NNK (4-(methylnitrosamino)-1-(3-pyridyl)-1-butanone), one of the many tobacco carcinogens, is able to upregulate the production of the chemokine CCL20 (C-C motif chemokine ligand 20); its production was inhibited by the anti-inflammatory drug dexamethasone, thus suppressing lung cancer in vitro and in vivo [59]. Furthermore, Shiels et al. conducted a nested case–control study where they demonstrated that the serum levels of some circulating inflammation markers, such as CXCL13 (C-X-C motif chemokine ligand 13), CRP (C-reactive protein), CCL22 (C-C motif chemokine 22) and IL-1RA (interleukin-1 receptor antagonist) were associated with increased lung cancer risk and offered a good separation in 10-year lung cancer cumulative risk between former smokers and current smokers [60].

Interestingly, it has also been demonstrated that lung inflammation derived from cigarette smoke is able to convert disseminated, dormant cancer cells to actively growing metastasis [61], thus suggesting that tobacco smoke can stimulate the recurrence of other cancer types than lung cancer. 

Cigarette smoke is not only associated with lung cancer but it also represents a risk factor for urothelial bladder cancer. In a retrospective analysis of 465,000 patients in the USA, it has been shown that there was a significant increase in cancer risk in both males and females, and also in former smokers [62]. This data was confirmed by Cumberbatch et al. who performed a meta-analysis on 88 studies to stratify the risk of bladder cancer among current smokers, former smokers and non-smokers [63]. Furthermore, smoking is also associated with a more aggressive phenotype and a higher risk of recurrence [64,65,66].

E-cigarettes are wrongly considered less harmful than tobacco cigarettes. However, their aerosol can also induce a considerable oxidative and inflammatory response in the lungs; in bronchial epithelial cells, e-cigarettes can induce acute toxicity and reduce the antiviral response [67,68]. Their users have high levels of neutrophil elastase, proteinase 3, azurocidin 1 and myeoloperoxidase, as well as other secondary neutrophil granule proteins in their sputum. All of these proteins are related to the innate defense role of leukocytes, bronchial inflammation and structural damage [69,70]. These results suggest that e-cigarettes, considered the safe alternative to tobacco cigarettes, can also induce lung inflammation and could therefore cause lung cancer. 

### 2.2. Ultraviolet Light 

Ultraviolet (UV) radiation is a part of solar radiation with shorter wavelength than visible light but longer than X-rays, and has been identified as a Group I type of carcinogen. Although moderate levels of UV exposure for a limited time are favorable for humans, since it stimulates the production of Vitamin D in our skin, an overexposure can cause skin inflammation (i.e., sunburn) and/or skin damage and skin cancer. Squamous cell carcinoma (SCC), basal cell carcinoma (BCC) and melanoma are the three main types of skin cancer; all of them can be triggered by UV exposure, especially in people with fair and sensitive skin [4,5,71,72]. UV radiation is commonly classified as UV-A (λ = 400–315 nm), UV-B (λ = 315–280 nm) and UV-C (λ = 280–100 nm). UV-C holds the highest energy and is therefore the most detrimental. Earth’s atmosphere is able to filter out solar UV-C and 90% of UV-B. UV-A is the main UV radiation able to reach Earth, representing 95% of the total UV radiation on the Earth’s surface. Nevertheless, UV-B, representing only 5% of solar UV radiation on Earth, is responsible of most of the health damage [73]. UV radiation can directly induce DNA damage and generation of ROS, prostaglandins, histamine and pro-inflammatory mediators, which are all able to alter immune cell functions. 

UV-B radiation can stimulate different skin cell types to produce inflammatory cytokines/chemokines. In particular, it has been demonstrated that both keratinocytes and Langerhans cells can secret cytokines, such as TGF-β, TNF-α; growth factors like PDGF (platelet-derived growth factor), NGF (nerve growth factor), bFGF (basic fibroblast growth factor) and CSF-1 (colony stimulating growth factor 1); and interleukins, such as IL1β, IL-6, IL-8, IL-10 and IL-12 (interleukin-12). UV-B exposure can therefore modify the secretion of these molecules and promote the inflammatory response. In particular, upon exposure to sunlight, these radiations stimulate keratinocytes to secrete pro-inflammatory cytokines (i.e., ΤΝF-α, IL-1α (intereleukin-1α), IL-1β, IL-6, IL-8 and IL-10), which are able to generate skin inflammation [74,75,76]. Several studies showed that ΤΝF-α, IL-1β and IL-6 stimulate macrophage migration and fuel inflammation at the exposed site [77,78,79,80]; IL-8 promotes tumors and metastasis in primary cutaneous melanoma after UV irradiation, while IL-33 (interleukin-33), known to promote angiogenesis, can be stimulated by inflammatory UV-B radiation [81,82]. Keratinocytes can also be stimulated to produce CXCL8, promoting the proliferation and migration of cSCC (cutaneous squamous cell carcinoma) cells, suggesting an important role of the CXCR1/CXCR2 (C-X-C motif chemokine receptor 1/2) axis in UV inflammation and tumorigenesis [83].

Moreover, Sung et al. demonstrated that skin cells, upon UV exposure, increase the production of prostaglandin and the release of histamine: mice deficient in prostaglandin E2 receptors are less susceptible to chemically-induced tumor formation [84]. Furthermore, COX1/2 (cytochrome c oxidase 1/2), which are enzymes involved in prostaglandin formation, are commonly present in UV or chemical-induced skin inflammation models [85,86]. Bald et al. showed that UV irradiation not only triggers tumor-initiating DNA alterations in melanocytes, but also stimulates their metastatic spread through TLR4 (toll-like receptor 4)/MyD88 (myeloid differentiation primary response 88)-driven neutrophilic inflammation originating from HMGB1 (high mobility group box 1) release from UV-damaged keratinocytes [87]. All these findings support the idea that inflammation is a pivotal process in UV-induced skin cancers. 

### 2.3. Asbestos Fibers

**Figure 3:**
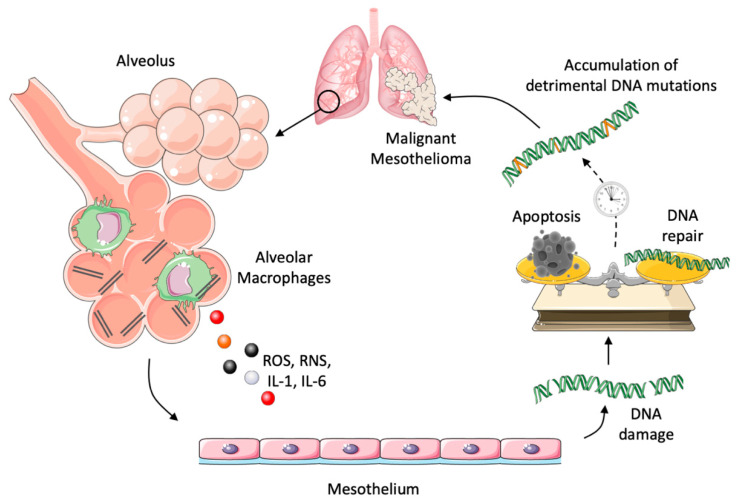
Overview of inflammation-driven mesothelioma development. Upon inhalation of asbestos in the lung, alveolar macrophages start a “frustrated phagocytosis” in the attempt to clear the non-degradable fibers away. Macrophages produce reactive oxygen and nitrogen species (ROS/RNS), as well as inflammatory cytokines (IL-1β, IL-6), which induce DNA damage, genetic alterations and inhibition of DNA repair mechanisms in mesothelial cells. The inflammation-induced tumorigenesis may need several years (black dotted arrow), since it is the result of a balance between accidental mutations and efficient DNA repair, mesothelial cell death or proliferation, and identification by or escape from the immune system. This figure was made with Servier Medical Art templates, which are licensed under a Creative Commons Attribution 3.0. Unported License (https://smart.servier.com).

Malignant mesothelioma is one of the clearest examples of a tumor arising as the result of chronic, decades-long inflammation (Figure 3). It develops from the mesothelial cells of the pleura (malignant pleural mesothelioma, MPM), the peritoneum of the pericardial cavity (typically affecting the lining of the lungs) and, less frequently, the lining of the abdominal cavity or heart [88]. Its occurrence is strongly related to professional or environmental exposure to particular carcinogenic fibers such as asbestos and other natural minerals with related chemical/physical properties (NOA, naturally occurring asbestos) [89,90,91]. The inhalation of asbestos fibers in the lungs stimulates long-lasting reactive inflammation in macrophages and in mesothelial cells, which is non-resolving, due to the non-degradable properties of these fibers [92,93]. Over several years, this unleashed inflammation causes DNA damage, accumulation of DNA mutations and, ultimately, neoplastic transformation [94].

Genetic mutations have been widely investigated in MPM. Overall, a comprehensive range of several mutations have been found in different genes, such as BAP1 (BRCA1 associated protein 1), CDKN2A (cyclin-dependent kinase inhibitor 2A), Ras (rat sarcoma protein), Wnt (wingless-related integration site), TP53 (tumor protein P53), SMARCB1 (SWI/SNF-related, matrix-associated, actin-dependent regulator of chromatin, subfamily B, member 1), NF2 (neurofibromin 2) and PIK3CA (phosphatidylinositol-4,5-bisphosphate 3-kinase catalytic subunit alpha) [95]. This variety of gene mutations in mesothelioma indicates that there are no specific driver mutations. 

In particular, it has been revealed that asbestos fibers stimulate the production of ROS/RNS both directly by mesothelial cells and by the recruited inflammatory macrophages [96,97,98]. Reactive oxygen and nitrogen species can therefore induce DNA damage, genomic instability such as DNA strand breaks and base modification, or alterations in DNA repair enzymes [99,100]. Accordingly, Marczynski et al. demonstrated an higher incidence of DNA double-strand breaks in the blood of workers occupationally exposed to asbestos [101].

Interestingly, Yang et al. [102,103] reported that asbestos is able to induce mesothelial necrotic cell death and the secretion of HMGB1. The release of HMGB1 induces macrophages to secrete TNF-α, which, in turn, activates NF-κB, which is responsible for the survival of some injured mesothelial cells. Pleural cells, that are able to survive to the initial asbestos injury, are repeatedly exposed to oxidative stress and inflammatory mediators, becoming predisposed to neoplastic transformation [102,103]. Furthermore, it has been demonstrated that asbestos fibers can directly activate the NLRP3 (NLR family pyrin domain containing 3) inflammasome in innate immune cells, triggering the production of active IL-1β and IL-18 (interleukin-18) in the microenvironment [104,105]. IL-1β can therefore activate the MyD88–IRAK (Interleukin 1 Receptor Associated Kinase 1)–NF-κB pathway, finally stimulating the secretion of secondary inflammatory mediators, such as TNF and IL-6. Hillegass et al. further demonstrated that the increase in these interleukins was blocked using the IL-1R antagonist Anakinra, both in vitro and in vivo [104]. Interestingly, Kadariya et al. [106] showed a significantly delayed tumor onset and reduced mesothelioma incidence in inflammasome-deficient mice (Asc mice), compared with wild-type animals chronically exposed to asbestos. The link between inflammation and the development of malignant mesothelioma provides the rationale for the use of inflammation-targeting therapies in mesothelioma patients, together with chemoprevention strategies targeting IL1β/IL1R signaling in the asbestos-exposed population [106].

## 3. Lifestyle

### Dietary Habits and Alcohol Consumption 

Diet plays a central role in health, being able to influence both the microbiota composition and to regulate the host’s physiological responses. The so-called “Western Diet” (WD) is characterized by high consumption of proteins (mostly animal-derived), saturated fatty acid, sugar, salt, alcohol, processed food and refined grains; at the same time, the WD is described by a reduced intake of vegetables, whole grains, vegetable-derived proteins, fruits, vitamin, minerals and omega-3 (n-3) polyunsaturated fatty acids (PUFAs) [107]. The WD has been associated with an increased risk of inflammatory bowel disease (IBD) [108] and with the development of cancer, type 2 diabetes, obesity, metabolic syndrome and cardiovascular diseases [109,110]. 

This kind of dietary habit is associated with high levels of serum markers of inflammation, thus suggesting its direct or indirect role in stimulating the immune system. In particular, it has been shown that the WD in mice is able to induce intense dysbiosis, with a strong reduction of commensal bacteria and an outgrowth of pathobionts. These microbiota changes are able to deeply influence the immune system through different ways, such as modification of signaling via the NLRP6 (NLR Family Pyrin Domain Containing 6) inflammasome and TLRs, the decrease of AMP (Adenosine Monophosphate) and mucus release in the lumen, the degradation of secretory IgAs (immunoglobulin A) and the selective loss of Treg lymphocytes producing IL-10. All these changes compromise barrier integrity and modify the intestinal immune cell homeostasis, leading to the onset of immune-mediated inflammatory disorders [111,112,113]. IBD patients have been shown to have an unbalanced enteric microbiota, with a decrease in anti-inflammatory commensals (e.g., *Faecalibacterium prausnitzii*), and an increase in pro-inflammatory Enterobacteriaceae (e.g., *Escherichia coli*) [114,115,116]. 

Moreover, an high intake of saturated fatty acids (SFAs) not only contributes to a state of metabolic endotoxemia by increasing the proportion of Gram-negative bacteria, but it is also able to activate a pro-inflammatory response in macrophages by stimulating a TLR4-induced inflammatory pathway; this pathway leads to the activation of NF-κB and stimulates the expression of other pro-inflammatory molecules [117,118]. Wrong dietary habits, such as WD, finally lead to overweight and obesity, thus promoting chronic low-grade systemic inflammation [119]. Adipose tissue is highly infiltrated by NK cells, mast cells, neutrophils, dendritic cells and macrophages [120]. In normal-weight people macrophages show an M2-like anti-inflammatory phenotype, while in obese people, they show an M1-like pro-inflammatory pattern that is able to produce tumor-promoting cytokines and chemokines, such as TNF, IL-6, IL-1β, CCL2 (C-C motif chemokine ligand 2) and MIF (macrophage migration inhibitory factor) [121,122]. Day et al., using a murine model of intestinal tumorigenesis, demonstrated that a high-fat diet, closely resembling the human WD, was able to alter the expression of macrophage markers and inflammatory mediators both in the adipose tissue and in the tumor microenvironment, thus increasing colorectal cancer (CRC) tumorigenesis [123]. Interestingly, Wunderlich et al. [124] revealed another mechanism able to connect diet-induced inflammation and CRC; their study demonstrated that diet-induced obesity accelerates chemically induced colitis-associated colorectal cancer (CAC) in mice by promoting inflammation and immune cell recruitment. In particular, they showed that IL-6 production can shape macrophages towards a tumor-promoting phenotype, which stimulates lymphocyte recruitment through the CCL20/CCR6 (C-C Motif Chemokine Receptor 6) axis, finally leading to CAC development [124]. 

Ultimately, it has been demonstrated that obesity is associated with the production of ROS, which actively contribute to cancer promotion [125]. Hyperglycemia and high levels of free fatty acid induce ROS production and the secretion of pro-inflammatory cytokines, leading to mitochondrial and DNA damage [126]. Accordingly, non-steroidal anti-inflammatory drugs have been linked with reduced cancer risk in obesity-related malignancies [127,128,129]. 

Chronic liver disease (CLD) can arise both from chronic viral Hepatitis B/C infection, and also from wrong dietary habits and alcohol consumption [130]. The WD brings to chronic and systemic inflammation that also affect the liver, where it can cause the non-alcoholic fatty liver disease (NAFLD) [131]. On the other hand, excessive alcohol consumption can cause alcoholic liver disease (ALD), which leads the liver to become inflamed and swollen. Although NAFLD and ALD are different diseases, both of them arise from the activation of inflammatory pathways within the liver [132]. The inflammatory activation of the hepatic stellate cells (liver-resident fibroblasts) leads to fibrosis, which eventually precedes the onset of the end-stage of liver disease: cirrhosis [133]. Cirrhosis is the greatest risk factor for the development of hepatocellular carcinoma (HCC), the sixth most commonly diagnosed cancer worldwide [134]. Moreover, 90% of the diagnoses of HCC occur in the context of chronic liver disease, underlying once again that chronic inflammation is a hallmark of this tumor [135]. It has also been demonstrated that high levels of alcohol-induced inflammation are significantly associated with disease severity and poor prognosis in HCC patients [136,137]. 

The Western diet and alcohol abuse lead to the development of hepatic steatosis and lipotoxicity (excessive accumulation of free fatty acids) within hepatocytes, resulting in modifications of membrane composition, mitochondrial dysfunction, generation of ROS, endoplasmic reticulum stress and the unfolded protein response [138]. All of these events finally lead to DNA damage and hepatocyte cell death, which stimulates the infiltration of immune cells [139]. The consequential chronic inflammation can further promote hepatocyte death, compensatory proliferation and fibrosis. In the end, these processes bring to HCC development [140]. 

## 4. Infection-Related

Around 15% of worldwide cancers arise as a direct consequence of infections [10,141]. Some infections are able to directly induce oncogenic events, such as Epstein–Barr virus (EBV) and human Papillomavirus (HPV): it has been shown that they are able to transform cells by inserting active oncogenes into the host genome. Although their presence is a necessary condition to induce tumor formation, it is not sufficient, and immune suppression is needed for tumor promotion [142,143,144]. The induction of infection-related tumor onset can also be enhanced by the following mechanisms: (i) viral genes are recognized by host cells and they induce the DNA damage response (DDR), increasing the genetic instability; (ii) viruses are able to promote chronic inflammation by generating reactive oxygen and nitrogen species, normally used by leukocytes to fight infection [9]; (iii) some infections are responsible for immune depression in some patients, disrupting normal immunosurveillance [26,145]. These events lead cells to chronic infection and, as a consequence, to chronic inflammation and cancer promotion.

### 4.1. Helicobacter pylori and Gastric Cancer

*Helicobacter pylori* (*H. pylori*) is a Gram-negative bacterium discovered in 1982 that is able to colonize the human stomach and is found in nearly half of worldwide population [146,147]. 15% of infected people develop gastric ulcers that can lead to gastric cancer [148,149]. The International Agency for Research on Cancer included it as Class I carcinogen [150]; in fact, *H. pylori* has been found to be associated with 60% of gastric cancers [151,152]. The mechanism of *H. pylori* infection begins with its penetration into the mucus layer and with the colonization of the mucosa, where it produces ammonia and carbonate ions, which induce a higher pH and reduce gastric acidity [153]. Machado et al. suggested that DNA damage can be caused by a downregulation of the repair processes [154]; this hypothesis has been confirmed by several research groups. Indeed, it has been demonstrated that gastric inflammation caused by *H. pylori* infection impairs mismatch repair mechanisms, via reduction of RNA levels of MutS (Mutator S) and MutL (Mutator L) [155,156]. Moreover, the increase in oxidative stress, as consequence of infection, causes DNA damage in epithelial cells [157,158]. Another mechanism has been revealed by Hudson et al. [159], who demonstrated that the expression of macrophage migration inhibitory factor (MIF) from macrophages and T-lymphocytes is able to suppress p53 transcriptional activity. This event is able to enhance proliferation and create an environment prone to accumulate potential oncogenic mutations [159].

The virulence factors of *H. pylori* are also associated with its role in the onset of gastric cancer: CagA (cytotoxin-associated gene A), VacA (virulence factor vacuolating cytotoxin A) and peptidoglycan induce an upregulation of several pro-inflammatory cytokines. Among these, IL1, IL-6, IL-8 and TNF-α are able to activate the NF-κB pathway, leading to leukocytes activation and to the release of several reactive species, such as peroxynitrite, a mutagenic agent [9]. As described above, the process of tissue repair in the presence of ROS and RNS, produced by inflammatory cells, mutates DNA. One of the observed mutated gene is p53, which has been shown to be mutated both in tumors and in chronic inflammatory diseases, such as inflammatory bowel disease [160], a predisposing condition for colon carcinogenesis.

### 4.2. Viral Hepatitis and Hepatocellular Carcinoma

As mentioned above, HCC is the sixth most commonly diagnosed cancer worldwide; this tumor is characterized by cirrhosis in 80% of cases and it was found to be associated to HBV (Hepatitis B virus) and HCV (Hepatitis C virus) in 54% and 31% of the patients, respectively [161,162,163,164]. These viruses belong to different families: HBV is a member of the Hepadnaviridae, while HCV is part of the Flaviviridae family. Only HBV has a direct effect on host cell DNA; in fact, cirrhosis was not found in all the HBV-positive tumors [165]. In contrast, the tumor-promoting effect of HCV is linked only to its ability to induce chronic inflammation [166]. 

Common mechanisms of action used by hepatitis viruses involve both viral and immune agents: several viral proteins are able to deregulate the hosts’ signaling pathways; moreover, the non-resolving inflammation caused by the viral infection results in immune-mediated stress damage, which leads, together with viral proteins, to intracellular oxidative stress [166]. 

Focusing on HCV infection, progressive liver fibrogenesis is caused by chronic hepatic inflammation and this event leads to cirrhosis development [167]. The onset and development of HCC arise after some decades of chronic inflammation associated with cirrhosis, pointing out the central role of inflammation in its pathogenesis. 

A key role in sensing HCV infection involves NF-κB signaling and its downstream pro-inflammatory chemokines and cytokines, such as Type III interferon (IFN) [168,169,170]. Finkin et al. demonstrated that, in a subset of HCV-infected human livers, NF-κB activation was associated with HCC initiation in an ectopic lymphoid structure aggregated near the portal tract [171]. An in vivo study demonstrated that ectopic lymphoid-like structures within the liver form an immunopathological microenvironment, which possibly serves as a niche to promote HCC initiation [171]. 

Another mechanism of tumor promotion is linked to the interaction between HCV core proteins and STAT3 protein, a molecule involved in the regulation of cytokine signaling [172,173]. The oxidative stress also enhances the aberrant deposition of extracellular matrix proteins and progressive fibrosis, turning the functional liver parenchyma into a non-functional fibrotic tissue. All together, these mechanisms create an immunosuppressive and permissive environment for hepatocarcinogenesis [174,175,176].

Recently, the introduction of direct-acting antivirals (DAAs) in the treatment of HCV led to virus elimination and to the consequent reduction of HCC [177]. Several groups also found that therapies based on IFN (interferon) were associated with a reduction in HCC risk [178]. Despite the incredible results of DAA treatment in the erasing of HCV viral titer, less is known about the effect on HCC onset and recurrence. In fact, until now, no evidence of a different HCC occurrence or recurrence has been found in patients treated with DAA and IFN therapy; probably the reduced risk is mainly associated with a sustained virologic response. In this post-HCV era, thanks to DAA therapy, HCC surveillance is currently recommended in all patients with cirrhosis [179]. HBV is not curable, but it has been almost eradicated after vaccine introduction; however, it remains a problem in those countries in which the vaccine is still not available, such as Eastern Asia. The tumor onset mechanisms of HBV are two: on the one hand, HBV DNA is able to integrate directly into the host DNA; on the other hand, HBV is able to establish chronic infection [166]. The chronic hepatitis phase is characterized by the increased adaptive immune response and a reduction of HBV DNA levels; as a consequence, there is a release of ROS and cytokines that induces immunosuppression. Taken together, these events lead to the development of fibrosis, cirrhosis and cellular transformation [180,181]. Since no drugs are available to treat HBV, several groups are currently studying the possibility of treating HCC induced by HBV chronic infection, with anti-PD-1/PD-L1 (programmed death-ligand 1), but the results are still not convincing [182].

### 4.3. HIV- and AIDS-Related Cancer 

The human immunodeficiency viruses (HIV) are two species belonging to the *Lentivirus* genus and they are members of the Retroviridae family. The disease derived from HIV infection causes the failure of immune system and is therefore named “acquired immunodeficiency syndrome” (AIDS), allowing both opportunistic infections and blooming tumors [183,184]. HIV has a tropism for cells of the human immune system, such as macrophages, dendritic cells and T-lymphocytes. HIV infection, through different processes, leads to the reduction of CD4+ T-cells to a critical level. Below this level, cell-mediated immunity is lost and this event allows the rise of opportunistic infections and AIDS development. HIV infection is associated with an increased risk of tumors, due to its ability to induce immunodepression [185,186]. Cancers related to HIV, classified as AIDS-defining cancers (ADCs), are induced by the reactivation of different viruses: Kaposi’s sarcoma from the re-activation of human herpesvirus 8 (HHV-8), non-Hodgkin lymphoma from EBV and invasive cervical cancer from HPV; their incidence is higher among HIV+ persons with advanced immunosuppression. Fortunately, highly active antiretroviral therapy (HAART) is able to reduce ADCs via viral suppression and immune restoration [187]. However, the incidence of other tumors is still under investigation. For these kinds of tumors, HAART does not seem to be associated with diminishing tumor incidence [187]. 

### 4.4. Other Pathogens

In this section, the role of parasites in tumor onset will be elucidated. Cholangiocarcinoma (CCA) and bladder cancer frequency are higher in subjects infected with *Opisthorchis viverrini*, *Clonorchis sinensis* and *Schistosoma haematobium* [188,189]. *O. viverrini* and *C. sinensis* are flatworms that colonize the bile ducts and infect humans; they are endemic in Eastern Asia and they have been identified as strong risk factors for CCA [188], being therefore classified as “carcinogenic to humans” [190]. In these parasite infections, the mechanism of CCA induction is linked to chronic inflammation that leads to cholangitis and fibrosis [191]. Pak et al [192] demonstrated in vitro that CAA cells (HuCCT1) treated with ESPs (excretory–secretory products) or the recombinant bacterial proteins of *C. sinensis* differentially produce pro-inflammatory cytokines (IL-1β, IL-6, and TNF-α) and anti-inflammatory cytokines (IL-10, TGF-β1, and TGF-β2). This evidence suggests that *C. sinensis* ESPs can contribute to the immunopathological response in host cells [192]. 

*S. haematobium* infection is considered a risk factor for bladder cancer due to the resulting inflammation that generates carcinogens, such as N-nitrosamines and DNA-damaging free radicals [193]. The sole use of the anti-helminthic agent praziquantel allow us to avoid both the infection and the consequent inflammation.

Another tumor that seems to be caused by pathogen infection and chronic inflammation is breast implant-associated anaplastic large cell lymphoma (BIA-ALCL) [194]. Lymphomagenesis may be derived from a chronic inflammatory reaction induced by both the capsule contents and surface material [195,196]. In particular, Hu et al. [196] found that the bacterial biofilm on breast implant capsules associated with ALCL has a different composition compared with that on non-tumoral implant capsules. They showed that the BIA-ALCL microbiome has a significantly greater proportion of *Ralstonia* spp. Gram-negative bacilli compared with the predominant *Staphylococcus* spp. observed in non-tumoral capsule tissue [196]. Moreover, it has been proven that the roughest textured surface prostheses represents a significantly greater risk compared with those with less prominent texturing [197,198], probably because bacterial growth and attachments are enhanced on the rough surface [199]. The presence of bacterial populations does not seem to depend on incorrect surgical practice, but it can be related to individual susceptibility and genetic host factors [200]. The repeated antigenic stimulation results in T-cell activation and chronic inflammation, via STAT3 signaling pathways [201,202]. 

## 5. Conclusions

The immune system is usually associated with defense against pathogens and external agents; however, it is now well accepted that they can also participate in tumor onset and progression. While the intrinsic pathway is mainly due to direct mutations of normal cells infected by viruses or other pathogens, the extrinsic pathway promotes the neoplastic process via inflammation. The inflammatory environment induces genomic alterations, favors angiogenesis and enhances tumor promoters. Moreover, tumor growth and migration are stimulated by cytokines, chemokines and reactive species, such as ROS and RNS, produced by both cells affected by chronic inflammation and newborn neoplastic cells. Several tumors are associated with chronic inflammation that can be derived from external agents, lifestyle habits or from infectious diseases (Table 1). Some of these tumors could be avoided thanks to prevention (e.g., UV protection, HBV vaccines, and healthy and balanced nutrition) and also current new therapies, such as DAA for HCV treatment, are all powerful weapons against inflammation-derived tumors. Recent immunotherapy studies must be further improved to bend the “dark side of the force” within the tumor and let the “light side” fight the enemy.

## Figures and Tables

**Table 1:** Chronic inflammation is associated with different tumor types.

Infectious or Inflammatory Agents	Cancer Type	Mechanisms	References
Environmental Factors			
*Tobacco smoke*	Lung cancer	ROS, chronic airway inflammation	[49,51,52,53]
	Bladder cancer		[59,62,63,65]
*E-cigarettes*	Lung cancer (potential)	Bronchial inflammation, structural damage	[69,70]
*Ultraviolet light (UV-B)*	Squamous cell carcinoma	ROS, prostaglandins	[84]
	Basal cell carcinoma	Skin inflammation	[74,75,76]
	Primary cutaneous melanoma	IL-33	[81,82]
*Asbestos fibers*	Mesothelioma	Chronic inflammation	[92,93,94]
		ROS/RNS	[99,100]
		HMGB1	[102,103]
		NLRP3 inflammasome	[104,105]
Lifestyle			
*Dietary habits and alcohol consumption*	Colorectal cancer	Dysbiosis and Chronic low-grade systemic inflammation (IL-6-CCL20/CCR6 axis)	[123,124]
	Hepatocellular carcinoma	ROS	[128,129]
Infection-Related			
*Helicobacter pylori*	Gastric cancer	Downregulation of repair mechanisms, oxidative stress, MIF	[154,157,158,159]
		CagA, VacA → inflammation	[9]
*HCV*	Hepatocellular carcinoma	Chronic inflammation (cirrhosis). NF-κB, STAT3, oxidative stress	[168,169,170,171,172,173,178]
*HBV*		Chronic infection and ROS	[166,181]
*HIV*	Kaposi’s sarcoma	Immunodepression→ Reactivation of HHV-8	[185,186]
	Non-Hodgkin lymphoma	Immunodepression→ Re-activation of EBV	
	Cervical cancer	Immunodepression→ Reactivation of HPV	
*Other pathogens*	Cholangiocarcinoma	*O. viverrini* and *C. sinensis*→chronic inflammation	[188,191,192]
	Bladder cancer	*S. haematobium→* N-nitrosamines and free radicals	[193]
	BIA-ALCL	Capsule contents/surface material→inflammation	[194,196,201,202]
